# A National Exploration of Medical Students’ Views Surrounding Skin Color Terminology in Undergraduate Education

**DOI:** 10.2196/92420

**Published:** 2026-07-14

**Authors:** Niamh Theresa McSwiney, Eliza Hutchison, Stephanie Lax, Paul Leighton, Hannah Wainman

**Affiliations:** 1 Bristol Medical School, University of Bristol, 5 Tyndall Avenue, Bristol, United Kingdom, 44 117 331 1831; 2 Department of Acute Medicine, University Hospitals Dorset NHS Foundation Trust, Castle Lane East, Bournemouth, Dorset, England, BH7 7DW, United Kingdom; 3 Centre for Applied Excellence in Skin and Allergy Research (CAESAR), University of Bristol, Bristol, United Kingdom; 4 Department of Dermatology, Royal United Hospital Bath NHS Trust, Bath, United Kingdom; 5 Lifespan and Population Health, School of Medicine, University of Nottingham, Nottingham, United Kingdom; 6 Department of Dermatology, University Hospitals Bristol and Weston NHS Foundation Trust, Bristol, United Kingdom

**Keywords:** skin of color, medical education, dermatology, medical student, terminology

## Abstract

We aimed to capture UK medical students’ preferences regarding skin color terminology in medical education and in relation to how they describe their own skin tone, to better understand how we can develop more inclusive, diverse medical language for both educational and clinical settings.

## Introduction

Many clinicians lack confidence recognizing and managing dermatological disease in patients with skin of color [1]. The clinical imagery and terminology surrounding skin color often present a narrow spectrum of individual experiences. For example, the Fitzpatrick Scale was developed in 1975 to classify reactivity of White skin to phototherapy but has now been adopted as a surrogate for describing skin color [2]. A public perspective on this topic has been considered, concluding that skin color scales should better reflect the lived experience and diversity of skin tones, particularly for darker skin, and descriptors should be meaningful to the public and not directly linked to race [3]. However, medical students’ voices remain lacking from these discussions. Medical students are the future of health care, medical education, and public health contributions. Therefore, their input can help shape more accurate, inclusive, and effective clinical training.

We aimed to capture UK medical students’ preferences regarding skin color terminology in medical education and in relation to how they describe their own skin tone, to better understand how we can develop more inclusive, diverse medical language for both educational and clinical settings.

## Methods

### Overview

A 16-question (multiple-choice and free-text) e-survey was created on Jisc Online Surveys and distributed to all UK medical schools with assistance from the Medical Schools Council, informed by a previous public survey [3]. The questions focused on views regarding the Fitzpatrick scale, descriptors used to describe respondents’ own skin, preferences for terminology when ethnicity is unknown, examples of inappropriate terminology in the educational setting, and subjective preparedness for clinical practice.

Survey distribution was standardized through a recruitment email, containing the study’s description, a participant information sheet, the author’s contact details, and a hyperlink to the online survey, disseminated to all academic year groups by each university, and remained open from October 1 to December 20, 2024. As dermatology teaching and clinical exposure vary between UK institutions in both timing and format, the authors included medical students from all academic year groups in survey participation to capture a broad, representative range of experiences. Free-text responses were not mandatory to promote survey completion. The survey was not piloted prior to nationwide distribution.

Quantitative responses were analyzed using descriptive statistics. Free-text answers were coded, categorized into themes, and analyzed manually using Microsoft Excel (version 26.108). All 367 responses were included in the final analysis.

### Ethical Considerations

A favorable ethnical opinion was granted by the University of Bristol Faculty of Health Sciences Research Ethics Committee (Ref 17622). No financial incentive was offered to the participants.

## Results

There were 367 responses from medical students studying at 22 medical schools (Table 1). Of 6 free-text questions, 1503 written responses were obtained (average response rate=68%, range 32%‐100%; response length: mean 6, range 1-127 words). Most respondents were aged 19‐24 years (69%) and female (71%)—slightly higher than the proportion of female medical school entrants nationwide (approximately two-thirds of entrants) [4]. Respondents were from diverse ethnic groups, with the ethnic distribution broadly similar to nationwide medical school demographics [4]. Participants were recruited from medical schools across the United Kingdom, with the largest proportions studying in the South West of England (22%), North East England (16%), and Scotland (13%).

Responses demonstrated that neither the pictorial nor text versions of the Fitzpatrick scale adequately represent how medical students consider their own skin, with 56% of them positioning themselves between or outside of the 6 skin tone images. Comments on the scale highlighted the need for greater representation of darker skin tones and consideration of other factors influencing skin color such as body site, ambient lighting, and sun exposure. On asking participants to describe their own skin color, the most frequent descriptors were brown (125 mentions), pale (n=123), and White (n=116). To describe their skin when inflamed or irritated, the most frequent terms were red (337 mentions), pink (n=97), brown (n=27), angry (n=22), and blotchy (n=20). The most common alternative descriptors for inflamed skin were bumpy/raised (95 mentions), dry/flaky/rough (n=91), and hot/warm (n=86). In situations when medical images are used without the individual’s ethnicity being known, the most frequently suggested alternative terminology was the objective skin color (28 mentions), such as “brown,” “Black,” or “White.” Other suggestions included the country of origin, descent, or heritage (n=19) and “light” or “dark” skin (n=7) of the person undergoing imaging. Medical students were asked whether they had perceived inappropriate terminology having been used in medical education, resulting in 48 comments. Thematic analysis generated 5 themes, which are summarized alongside representative quotes in Table 2. Many commented on the use of offensive or outdated terminology, such as the term “colored,” historical labels such as “Caucasian” or “Oriental” and the use of racial or ethnic terms as nouns such as “Blacks.”’ Several respondents commented on a lack of variation in descriptors and highlighted skin color as a spectrum rather than just “White or Black.” Generalizations such as “BAME” (Black, Asian and Minority Ethnic), “brown,” and “skin of color” were perceived as restrictive and unable to capture important differences. Many reported “White skin” and “redness” predominating in their education with limited exposure to diverse imagery and language. When skin tone was discussed by educators, some felt it was delivered in a “disrespectful” and “awkward” manner, and many still “struggle to find the right terminology with darker skin.”

When asked to rank their subjective preparedness for clinical practice using a 5-point Likert scale (1=not at all prepared and 5=extremely well-prepared), 265 (72%) responders reported feeling neutral to unprepared in relation to seeing patients with diverse skin tones and 274 (74%) reporting feeling neutral to underprepared in relation to discussing differences in the presentation of dermatoses in skin of color with patients.

**Table 1.** Characteristics of respondents (N=367).

Characteristics	Respondents, n (%)
Age (years)	
<18	43 (12)
19‐24	253 (69)
25‐34	60 (16)
≥35	8 (2)
Prefer not to say	3 (1)
Gender	
Female	261 (71)
Male	89 (24)
Other/prefer not to say	17 (5)
Ethnicity	
Asian or Asian British	110 (30)
Black, African, Caribbean or Black British	38 (10)
Mixed/Multiple Ethnic Groups	28 (8)
White	174 (47)
Other Ethnic Group	17 (5)
Location of the medical school	
England	
East of England	32 (9)
East Midlands	12 (3)
London	8 (2)
North East	57 (16)
North West	32 (9)
South East	41 (11)
South West	82 (22)
West Midlands	31 (8)
Scotland	49 (13)
Wales	23 (6)

**Table 2.** Key themes and representative quotes generated from survey.

Theme	Representative quote
Offensive and outdated terminology	“Colored - this has a dark history in relation to when the word came about and is unnecessary.” [Female, 19‐24, Black Caribbean] “I don’t like the term ‘Caucasian’ being used to describe people with white skin, like myself. It is outdated and comes from a racist, hierarchical naming convention and I would much rather be called white.” [Female, 25‐34, White] “Depends on the situation - some individuals dislike being called ‘colored’ or ‘Black,’ and other individuals feel that these terms are acceptable. I think it may depend on the individual’s experience and the behavior of the person who is referring to them (social environment, power imbalance).” [Male, 19‐24, Mixed White and Asian]
Lack of variation in descriptors	“Sometimes skin has been referred to as black or white (such as the current general terms for race). I think feel that medically these terms should not be used to describe skin color as this is factually incorrect. No skin is either white or black. There are various shades of browns, pink and cream.” [Female, 35‐44, Mixed White and Black African] “Explicitly white or black skin - neither of the skin colors are true representatives; paler or darker skin color would be more acceptable as skin color is a spectrum rather than direct category” [Female, 19‐24, Any other White background]
Generalizations	“I think the term BAME is outdated because it groups together all non-white patients when the differences between them in terms of treatment and outcomes can massively vary.” [Male, 19‐24, Mixed White and Asian] “Asian being used to cover East Asian, South Asian, and West Asian skin colors as a catch all, even though they are very different and not descriptive at all.” [Male, 19‐24, Asian Indian]
Representation in medical education	“Usually we are not exposed to many skin type images for conversations to even occur.” [Female, 19‐24, Asian Bangladeshi] “I find the use of redness to be a relic of white hegemony in medicine given that most people in the world are non-white. I have seen textbooks say conditions are harder to recognize in darker skin which is lazy and irresponsible given how many doctors abroad rely on them for medical training.” [Male, 25‐34, Black African]
Insensitive delivery	“I do think people struggle to find the right terminology with darker skin, often clinicians just want to be correct (politically and clinically).” [Female, 19‐24, White] “In another lecture black scientists were randomly discussed as if to show that there are a couple of black people who have contributed to science. It is well intentioned (supposedly) but awkward and passive aggressive.” [Female, 25‐34, Asian Indian]

## Discussion

This nationwide survey highlights the need for more inclusive, representative terminology for skin color in medical education. Tools such as the Fitzpatrick scale fail to reflect the full spectrum of skin tones, which is acknowledged in the literature [2]. More inclusive models are being developed, including The Eumelanin Human Skin Color Scale and the Monk Skin Tone Scale, and it is essential that these scales are adapted for and incorporated into medical education [5-7]. Aside from ethnicity, suggestions for preferred terminology were heterogeneous with objective color-based descriptors predominating, although these terms can underrepresent the full spectrum of skin tone variation. Despite recent efforts to diversify medical curricula, White-centric imagery and language still predominate, translating to many students still feeling underprepared seeing patients with diverse skin tones. When educators do discuss skin color, there are still instances of inappropriate, outdated terminology being used.

Limitations of our study include the small sample size and potential selection and recall bias. This study was not funded; hence, there was no financial incentive for medical students to participate. Incentives or more targeted recruitment strategies may have improved participation. However, to our knowledge, this is the first study exploring medical students’ opinions on this topic, with UK-wide experiences captured.

We highlight the importance of developing clearer, more descriptive skin color terminology with greater representation of diverse skin tones in medical curricula. Educators should consider the language they use to describe skin color and be open to conversations with students about appropriate terminology. Future work to develop a consensus about how we describe skin color in dermatology is essential, and it is important to consider the views of medical students as well as patients and clinicians [7]. This will not only support students’ learning but also foster more equitable dermatological care for patients with diverse skin tones.

## Abbreviations

BAME: Black, Asian and Minority Ethnic
